# Impact of NHS breast screening on advanced disease and mortality from breast cancer in the North West of England

**DOI:** 10.1038/sj.bjc.6600842

**Published:** 2003-07-01

**Authors:** A G Threlfall, S Collins, C B J Woodman

**Affiliations:** 1 Centre for Cancer Epidemiology, The University of Manchester, Kinnaird Road, Withington, Manchester M20 4QL, UK

**Keywords:** breast cancer, screening, mortality, advanced disease

## Abstract

A cohort study was undertaken to describe outcomes from breast cancer in women who were aged 54 years or younger when they were first invited for NHS breast screening. The analysis included 5125 women invited for multiple rounds of breast screening by the Wigan screening programme and 10 750 women invited by the Manchester programme. The main outcome measures were rates of advanced disease and mortality from breast cancer. In Wigan 4028 (78.6%) and in Manchester 5485 (51.0%) women accepted all of their invitations for screening. The incidence of invasive cancer was higher in Wigan than in Manchester (24.78 *vs* 21.11 per 10 000 person-years; *χ*^2^=2.11, 1 df, *P*=0.15), but the rate of advanced disease was significantly lower (2.49 *vs* 4.73 per 10 000 person-years; *χ*^2^=4.36, 1 df, *P*=0.04). Mortality was lower in Wigan than in Manchester (2.46 *vs* 4.31 per 10 000 person-years; *χ*^2^=3.25, 1 df, *P*=0.07). In the first report of long-term outcomes in women invited for NHS breast screening, we demonstrated that it is possible to evaluate the impact of screening by comparing programmes with different proportions of regular attenders; a significant difference was shown in the rate of advanced disease between two programmes with different cancer detection and attendance rates.

The NHS breast-screening programme was introduced in 1988 after the Swedish two-county trial demonstrated a substantial reduction in mortality from breast cancer in women who received repeated invitations for screening (Tabàr *et al*, 1985). The results of randomised controlled trials cannot always be replicated outside the trial setting, and the actual impact of NHS breast screening on breast cancer mortality needs to be determined. However, evaluating the national programme is problematic because of changes over time in treatment and in the underlying incidence of disease (Prior *et al*, 1996; Blanks *et al*, 2000a). Furthermore, mortality data are not routinely linked to screening histories, which can result in the misclassification of exposure to screening: for example, the failure to exclude deaths from cancers diagnosed before the introduction of screening dilutes the observed effect of the programme (Blanks *et al*, 2000a). In this study, we used record linkage to assemble individual screening histories, and relate these to the occurrence of advanced disease and death from breast cancer in a cohort of women who received multiple invitations to screening. This allowed us to compare outcomes in women attending two screening programmes with substantially different attendance rates during the same period.

## MATERIALS AND METHODS

The base population comprises 17 305 women who were aged 54 years or younger when they were first invited for screening by either the Manchester screening programme, between 1 January 1989 and the 30 September 1990, or by the Wigan screening programme, between 1 January 1989 and 30 June 1990. The study population was confined to women aged 54 years or younger because only these women had a screening experience comparable to that of the women who are now being invited to screening *for the first time,* and future comparisons between programmes will be restricted to women in this age range. Not all screening programmes in the North West region commenced screening in 1988, only the Manchester, Wigan, and Bolton programmes were able to participate at this time. By including only those programmes that began screening in 1988, the duration of follow-up was maximised and the confounding effect of secular trends in the diagnosis and management of disease were avoided. However, data from the Bolton programme could not be included because the method of data storage used initially by this programme did not allow the assembly of full screening histories. Women invited for screening during 1988 in Wigan and Manchester were not included to allow for the solution of any early problems which are inevitable with new systems.

All primary breast cancers occurring in this population were identified from records held at the North Western Regional Cancer Registry. To ensure that only residents of the North Western Region contributed to the analysis, records of all women invited for screening were linked with those held by the NHS Central Register. Excluded from further analysis were 113 women who had died or left the region before their first scheduled screen, 859 who had their registration with a general practitioner cancelled during follow-up because their whereabouts were unknown, 266 for whom no match was found on the NHS Central Register, and 192 women in whom breast cancer had been diagnosed before their first scheduled screen.

Person-years at risk were measured from the date of the first screen to which the woman was invited. Women were withdrawn from follow-up when they died or left the region; end of follow-up in the remaining women was 30 June 2000. Women who accepted an invitation to screening were classified as attenders and those who declined all invitations, nonattenders; women who declined their first invitation but attended a subsequent screening round contributed follow-up time to the nonattenders class until they first attended screening, after which they contributed follow-up time to the attenders class. Cancers were categorised according to mode of detection. In attenders, the categories were: first-screen cancers, if detected at the first screening round; rescreening cancers, if detected at a subsequent screening round; interval cancers, if diagnosed in the interval between screens and the woman had attended her previous screen, or lapsed attender cancers, if detected in the interval between screens and the women had failed to attend her previous screen. A cancer in a woman who had never attended screening was classified as a nonattender cancer.

Following a review of pathology reports and hospital records, stage at diagnosis was assigned by one of the authors (AGT) using the International Union Against Cancer classification of tumours (UICC, 1987). Women with stage III and stage IV disease, and those with four or more positive lymph nodes, were categorised as having advanced disease (Bundred *et al*, 2000; Miller *et al*, 2000). A woman was considered to have died from breast cancer, if this was recorded as the underlying cause of death in sections 1a, 1b, or 1c of the death certificate. To examine the impact of any population differences in the prevalence of advanced disease at the first screening round, incidence and mortality rates are reported both before and after the exclusion of women found to have cancer at this time. To determine the relative contribution of each mode of detection to the overall incidence, advanced disease, and mortality rates in each population, incidence and mortality rates are constructed using the total number of person-years accrued by all women invited for screening by each programme as the denominator. Statistical methods suitable for small sample sizes were used, as appropriate, for significance testing and interval estimation; all tests were conducted at the 5% two-sided significance level (Martin and Austin, 1996).

## RESULTS

The final study population includes 10 750 women invited by the Manchester programme and 5125 by the Wigan programme. A total of 9074 (84.4%) women in Manchester and 4724 (92.2%) in Wigan attended on at least one occasion (95% confidence interval for difference in proportions, 6.8–8.8%); 5485 (51.0%) women in Manchester and 4028 (78.6%) in Wigan accepted all screening invitations (95% confidence interval for difference in proportions, 26.1–29.1%). During follow-up, 223 and 129 invasive cancers were diagnosed in Manchester and Wigan, respectively; their size, stage, and nodal status are shown in Table 1

**Table 1:** Pathological classification of invasive breast cancer in Manchester and Wigan

		**Manchester**		**Wigan**	
**UICC stage**	**Pathological classification^a^**	**Number**	**%**	**Number**	**%**
I or II	*Tumour size ⩽20 mm*				
	Node negative	80	36	51	40
	1–3 positive nodes	14	6	15	12
	Four or more positive nodes	10	4	3	2
	Unknown nodal status	31	14	27	21
					
	*Tumour size 21–50 mm*				
	Node negative	20	9	12	9
	1–3 positive nodes	17	8	5	4
	Four or more positive nodes	11	5	3	2
	Unknown nodal status	6	3	4	3
	Size unknown	5	2	2	2
					
III or IV		29	13	7	5
					
Total		223		129	

a In nine (6%) cases in Manchester and nine (10%) cases in Wigan, nodal status is based upon less than four lymph nodes.

. A total of 50 women in Manchester and 13 in Wigan presented with advanced disease, of whom 33 (66%) and eight (62%) died during follow-up. Cancer detection rates were higher in Wigan than in Manchester in the first three screening rounds, but not in the fourth (Table 2

**Table 2:** Attendance and cancer detection rates at the first four screening rounds in Manchester and Wigan

**Round**	**Programme**	**Number invited^a^**	**Number attended (%)**	**Invasive cancers detected**	***In situ* cancers detected**	**Detection rate per 1 000 screens**
1	Manchester	10 750	7727 (71.9)	21	8	3.8
	Wigan	5125	4380 (85.5)	18	6	5.5
2	Manchester	10 212	7369 (72.2)	17	10	3.7
	Wigan	4936	4255 (86.2)	18	5	5.4
3	Manchester	9741	6931 (71.2)	29	6	5.0
	Wigan	4703	4157 (88.4)	20	5	6.0
4	Manchester	8118	5719 (70.4)	39	3	7.3
	Wigan	3969	3523 (88.8)	20	1	6.0

a In total, 288 women in Manchester and 164 in Wigan were invited to five or more screens.

).

The incidence of invasive cancer was higher in Wigan than in Manchester (24.78 *vs* 21.11 per 10 000 person-years; *χ*^2^=2.11, 1 df, *P*=0.146), but the incidence of advanced disease was significantly lower (2.49 *vs* 4.73 per 10 000 person-years; *χ*^2^=4.36, 1 df, *P*=0.04). The rate of advanced disease in nonattenders was similar in the two populations (7.48 *vs* 7.73 per 10 000 person-years; *χ*^2^=0.0035, 1 df, *P*=0.95) and higher than that in attenders. Mortality rates were lower in Wigan than in Manchester (2.46 *vs* 4.31 per 10 000 person-years; *χ*^2^=3.25, 1 df, *P*=0.072) and remained lower after exclusion from the analysis of women found to have cancer at the first screening round (2.28 *vs* 4.23 per 10 000 person-years; *χ*^2^=3.70, 1 df, *P*=0.054) (Table 3

**Table 3:** Incidence, advanced disease, and mortality rates in all invited women, in nonattenders, and in all invited women without cancer detected at the first screening round, in Manchester and Wigan

	**Manchester**			**Wigan**			
**Population**	**Person-years**	**Number of events**	**Rate per10 000 years**	**Person-years**	**Number of events**	**Rate per10 000 years**	**Risk ratio(95% CI)**
*All invited women*							
Incidence	105 649	223	21.11	52 050	129	24.78	0.85 (0.69–1.06)
Advanced disease	105 649	50	4.73	52 050	13	2.49	1.89 (1.05–3.62)
Mortality	106 784	46	4.31	52 773	13	2.46	1.75 (0.96–3.35)
							
*Nonattenders*							
Incidence	20 696	37	17.87	5349	10	18.69	0.96 (0.49–2.02)
Advanced disease	20 696	16	7.73	5349	4	7.48	1.03 (0.36–3.60)
Mortality	20 857	15	7.19	5380	6	11.15	0.64 (0.26–1.81)
							
*All invited women excluding cancers detected at first screen*							
Incidence	105 649	202	19.12	52 050	111	21.33	0.90 (0.71–1.13)
Advanced disease	105 649	48	4.54	52 050	12	2.31	1.97 (1.07–3.86)
Mortality	106 489	45	4.23	52 538	12	2.28	1.85 (1.00–3.64)

).

The mode of presentation of all cancers and of advanced cancers, and the deaths that followed from them, are reported in Table 4

**Table 4:** Number and rate per 10 000 person-years of follow-up of invasive cancers, advanced cancers, and mortality from breast cancer according to mode of presentation in all women invited for screening by Manchester and Wigan

	**Invasive cancer**				**Advanced cancer**				**Mortality**			
	**Manchester**		**Wigan**		**Manchester**		**Wigan**		**Manchester**		**Wigan**	
**Mode of presentation**	***n***	**Rate**	***n***	**Rate**	***n***	**Rate**	***n***	**Rate**	***n***	**Rate**	***n***	**Rate**
First screen	21	1.99	18	3.46	2	0.19	1	0.19	1	0.09	1	0.19
Rescreening	87^a^	8.23	58	11.14	9	0.85	2	0.38	8	0.75	1	0.19
Interval	62	5.87	39	7.49	19	1.80	6	1.15	19	1.78	5	0.95
Lapsed attender	16	1.51	4	0.77	4	0.38	0	0.00	3	0.28	0	0.00
Nonattender	37	3.50	10	1.92	16	1.51	4	0.77	15	1.40	6	1.14
												
Total	223	21.11	129	24.78	50	4.73	13	2.49	46	4.31	13	2.46

a One cancer was detected in the fifth round and one cancer was detected by the Bolton screening programme.

. In Wigan, fewer women were nonattenders and therefore the contribution of cancers in nonattenders to the overall incidence of advanced disease was less than in Manchester. Furthermore, a greater proportion of women in Wigan attended all of their screens, so fewer cases of advanced breast occurred in women who lapsed from attending screening.

## DISCUSSION

Rates of advanced disease and mortality from breast cancer were lower in women invited for screening by the Wigan programme than by the Manchester programme, although the incidence of breast cancer was higher in Wigan. The lower mortality rate in Wigan, if not because of chance, must be because of a more favourable stage distribution, more effective treatment, or a combination of both. A more favourable stage distribution is the most likely explanation, because the rate of advanced disease was significantly lower in Wigan than in Manchester, and advanced disease had a poor outcome in both populations. Fewer women in Wigan had nodal surgery, which might suggest that the rate of advanced disease was underestimated there. However, the difference in the frequency of nodal surgery was confined to women with small screen detected tumours, most of which were detected at screening, and rarely have multiple positive nodes (Bundred *et al*, 2000). Therefore, the impact of differences in nodal surgery on the rates of advanced disease is likely to have been small. The rate of advanced disease might also be lower in Wigan than in Manchester, if unscreened women in Wigan presented earlier with symptomatic disease. However, the incidence of advanced disease in nonattenders and the prevalence of advanced disease at the first screening round were similar in the two populations. Therefore more effective screening, either as a result of higher detection rates or greater attendance at screening, is the most likely explanation for the lower rate of advanced disease in Wigan. Differences in attendance alone, however, cannot fully explain the difference in the overall rate of advanced disease between the two populations. Rescreening and interval cancers contributed more to the rate of advanced disease in Manchester than in Wigan, possibly because of the lower detection rates in the early years of the Manchester programme.

Although the magnitudes of the reduction in the incidence of advanced disease and mortality from breast cancer are coherent and consistent with a more effective screening programme in Wigan, the magnitude of the effect is greater than that which would have been predicted from the results of trials in which comparisons are made with a control group that is not invited for screening. We have no compelling explanation as to why this should be the case, and can only point out that, although the difference between the two programmes in the incidence of advanced disease is significant at the 5% level, the 95% confidence interval is wide, and is consistent with a relative risk reduction of as little as 7%.

The NHS breast-screening programme is a national programme that invites all women in the target age range. Therefore, it is not possible to evaluate the impact of NHS screening by comparing outcomes from breast cancer in women invited to screening with those in a contemporaneous control group of uninvited women of a similar age. However, this study demonstrates that it is possible to evaluate individual programmes using a cohort study design with record linkage. This is the first report of long-term outcomes from breast cancer in women invited for NHS screening and shows a significant difference in the rate of advanced disease between two programmes with different cancer detection and attendance rates. Cancer detection rates have improved throughout the NHS breast-screening programme and now commonly exceed those seen in Wigan during this study, but routinely collected statistics do not allow us to assess whether the high proportion of regular attenders observed in Wigan has been replicated elsewhere within the programme (Blanks *et al*, 2000b). If this proportion varies between individual programmes, then comparisons between programmes with different proportions of regular attenders may provide a means for evaluating the impact of NHS breast screening. The low rate of advanced disease observed in the Wigan programme indicates what can be achieved with high cancer detection rates and regular attendance at screening.
